# Mobility Matters: Differential Mobility Loss by Race and Ethnicity in Hawaiʻi

**DOI:** 10.5888/pcd23.250407

**Published:** 2026-05-07

**Authors:** Miquela Ibrao, Yan Yan Wu, Kathryn L. Braun

**Affiliations:** 1 Thompson School of Social Work and Public Health, University of Hawaiʻi at Mānoa, Honolulu, Hawaii

## Abstract

**Introduction:**

Mobility is a critical determinant of healthy aging. Agility, gait, balance, and fall risk, when left unassessed and unaddressed, may diminish older adults’ ability to age in place, often leading to more restrictive, supervised care environments. This study examined racial and ethnic disparities in a composite mobility/functional measure in Hawaiʻi and the associations of selected social determinants of health (SDOH) with limitation status.

**Methods:**

We analyzed data from the Hawaiʻi Behavioral Risk Factor Surveillance System collected from 2019 through 2021. The study population included community-dwelling adults aged 55 years or older from the 4 largest racial and ethnic groups in Hawaiʻi: White, Filipino, Japanese, and Native Hawaiian (unweighted n = 10,039; weighted population estimate = 350,922). We used weighted logistic regression to assess associations between mobility limitations and SDOH.

**Results:**

Mobility limitations were reported by 28% of Native Hawaiian people aged 55 years or older, compared with 17% to 19% among other groups. Native Hawaiian adults aged 55 to 64 years also had substantially higher prevalence of mobility limitations than adults of the same age in other racial and ethnic groups. Higher income was protective against mobility limitations for both Native Hawaiian and White adults. In contrast, the associations of education and health insurance with mobility limitations varied across groups, with weaker protective associations of education among Native Hawaiian adults.

**Conclusion:**

Findings suggest the importance of considering mobility-focused prevention and assessment for Native Hawaiian adults before the Medicare eligibility age of 65 years. To be effective, these interventions must be culturally grounded and tailored to the unique needs and lived experiences of Native Hawaiian communities.

**Summary**

**What is already known about this topic?**

Mobility is a key determinant of healthy aging, as declining mobility limits older adults’ ability to age in place.

**What is added by this report?**

We compared mobility limitations in older adults (aged ≥55 y) among Hawaiʻi’s largest racial and ethnic groups. Findings showed a higher prevalence of mobility limitations among Native Hawaiian adults than among White, Filipino, and Japanese adults, with disparities already evident among adults aged 55 to 64 years. Associations between selected social determinants of health and mobility limitations also differed across racial and ethnic groups.

**What are the implications for public health practice?**

Early assessment and culturally grounded interventions may help support mobility and reduce disparities among Native Hawaiian adults.

## Introduction

Mobility is a critical determinant of healthy aging and longevity, influencing a person’s ability to live independently, maintain social connections, and prevent chronic conditions. The human body’s natural aging process results in a loss of strength, muscle mass, agility, and balance that may restrict one’s ability to be mobile and to live independently (1). The prevalence of mobility limitation also increases with age; approximately 12% of US adults aged 18 years or older report a mobility disability (2), and mobility limitation is substantially more common among older adults (3).

Mobility limitations do not affect all racial and ethnic groups equally. For instance, studies in the US have found that racial and ethnic minority populations, such as Black adults, have higher mobility limitations than White adults (4,5). Hawaiʻi’s population is among the most racially and ethnically diverse in the US. According to 2023 Hawaiʻi population estimates, only 23% of the population was White, and about two-thirds was Asian or Native Hawaiian and other Pacific Islander (6). Asian subgroups in Hawaiʻi with relatively large populations include Japanese, Filipino, and Chinese. The state’s demographic complexity underscores the importance of disaggregating racial and ethnic categories, as aggregated data often conceal health disparities among subgroups, particularly in Native Hawaiian, Pacific Islander, and Filipino populations (7).

The social determinant of health (SDOH) framework, as defined by Healthy People 2030, encompasses 5 domains: education access and quality, health care access and quality, economic stability, neighborhood and built environment, and social and community context (8,9). These interrelated factors shape lifelong health outcomes and are powerful drivers of inequities in functional ability and mobility among older adults (10,11). The framework is an effective tool to explore and explain existing inequities in populations and can be used as a mechanism to approach change. Evidence-based strategies to promote mobility — such as increasing opportunities for physical activity and home-based rehabilitation — align closely with SDOH domains (12). Yet access to these interventions is often shaped by structural inequities. For example, safe walking spaces and recreational facilities are less available in low-income or rural neighborhoods, and home-based therapy services may be limited by workforce shortages and reimbursement barriers (13).

Despite national attention to mobility disparities, limited research has examined these patterns within the context of Hawaiʻi’s racially and geographically diverse population. A 2003 analysis of the Hawaiʻi Behavioral Risk Factor Surveillance System (BRFSS) found higher rates of activity limitations and mobility-device use among Native Hawaiians compared with Japanese and White residents (8). More recently, a 2021 study found greater functional limitation among Native Hawaiian and Pacific Islander, Black, and Hispanic populations than among White and Asian populations in Hawaiʻi (14); however, the authors did not disaggregate Asian populations. Disaggregation of races and ethnicities in the state is important because different Asian and Pacific Islander subgroups experience health disparities disproportionately (7). These disparities may reflect earlier onset of chronic disease and socioeconomic disadvantage, underscoring the need to examine mobility limitations among those younger than the traditional retirement age of 65, as well as to provide context for findings within SDOH domains.

For older adults living in Hawaiʻi, barriers to healthy aging in the community include diasporic populations spread across 7 islands. This distance often requires flights and overnight stays to see specialized health care providers, which are primarily concentrated in Honolulu on the island of Oʻahu. Individual islands also have fewer social agencies to provide comprehensive wraparound services, including transportation (15). The services that are present for older adults may be underused by them because of factors that include high cost of participation and lack of perceived cultural competency (16). Further research is warranted on mobility limitations at younger ages and on the role of SDOH in this population.

This study extends the literature by using recent (2019–2021) Hawaiʻi BRFSS data to examine mobility/functional limitations among community-dwelling adults aged 55 years or older, disaggregating major racial and ethnic groups and situating disparities within an SDOH framework. Prior studies in Hawaiʻi used older datasets, did not examine adults aged 55 to 64 years, or did not disaggregate racial and ethnic populations. We hypothesized that mobility limitations would differ significantly across racial and ethnic groups in Hawaiʻi, with higher prevalence among Native Hawaiian compared with White, Filipino, and Japanese populations. We also hypothesized that these disparities would be evident among adults younger than the traditional older-adult threshold of 65 years and would be associated with SDOH.

We examined differences in a composite mobility/functional limitation measure (defined by difficulty walking, climbing stairs, dressing, bathing, or running errands independently) across racial and ethnic groups among community-dwelling adults aged 55 years or older who participated in the Hawaiʻi BRFSS from 2019 through 2021. Understanding these disparities has implications for clinical practice and public health planning. Identifying populations with disproportionately high limitation prevalence at ages 55 to 64 years may guide earlier screening, culturally appropriate interventions, and targeted community-based services to support aging in place. These findings may also inform resource allocation, transportation planning, and preventive programs aimed at reducing disability and health inequities among older adults in Hawaiʻi.

## Methods

### Data source

This study used 3 annual waves of Hawaiʻi BRFSS data collected from 2019 through 2021. The BRFSS aims to collect uniform state-level data on health-related risk behaviors, chronic disease, and health care access, among adults (aged ≥18 years) in the US. Data are collected and managed by each state’s health department with guidance and oversight from the Centers for Disease Control and Prevention (CDC). The State of Hawaiʻi has been participating in the BRFSS since 1986 (17). The BRFSS survey sampling design uses a disproportionate stratified sample to call landlines and cellular telephones of households in Hawaiʻi. Only community-living adults are included in the sample; those who live in congregate living settings, such as shelters and long-term care facilities, are excluded from the survey. To reduce biases from unequal selection probability, the BRFSS adjusts design weights through an iterative proportional fitting process based on demographic and geographic margins, including age, sex, race and ethnicity, education, marital status, homeownership, and telephone type (18). Survey weights were iteratively adjusted across these variables until convergence to population margins, improving representativeness without requiring full cross-classification. This study was approved by the University of Hawaiʻi’s institutional review board and by the Hawaiʻi State Department of Health.

### Study sample and variables

The study sample included adults aged 55 years or older who responded to the BRFSS annual survey from 2019 through 2021 from the 4 largest racial and ethnic groups in Hawaiʻi: White, Filipino, Japanese, and Native Hawaiian. The combined Hawaiʻi BRFSS sample over the 3-year period included 23,224 respondents. We excluded respondents younger than 55 years (n = 10,951), respondents whose race or ethnicity was not included in this analysis (n = 1,786), and respondents with missing data on the outcome (n = 302). We also excluded respondents with missing or refused data for sex at birth, education, health insurance, marital status, or island of residence (n = 146). Household income and neighborhood walkability were the 2 variables with the highest amount of missing and refused responses, which were recoded as “unknown” and retained in the analysis (n = 1,950). After selecting data for the population of interest and excluding or recoding missing cases as unknown to maintain a sample size large enough to run analyses of association, the final unweighted sample size for the study was N = 10,039 (Figure).

**Figure:**
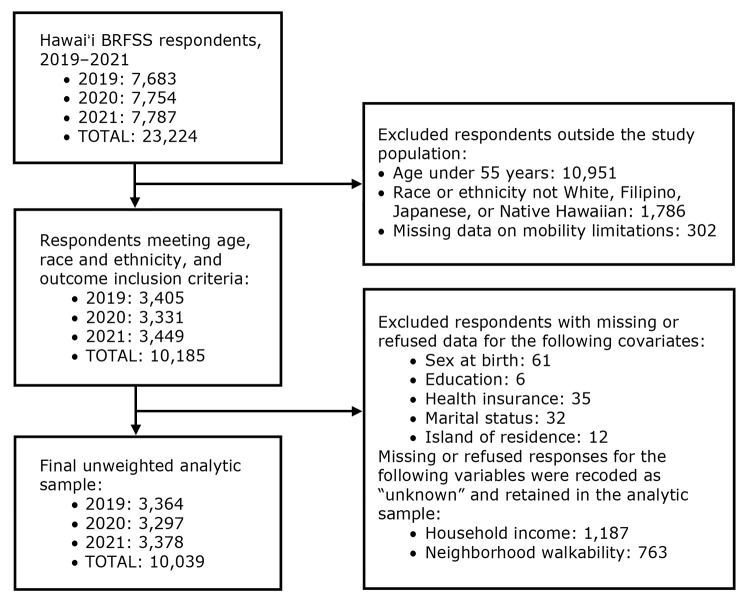
Derivation of the unweighted analytic sample from the Hawaiʻi Behavioral Risk Factor Surveillance System, 2019–2021.

The outcome of interest for this study was a dichotomous composite measure indicating 1 or more self-reported mobility/functional limitations. Responses were recoded into a single dichotomous dependent variable by using 3 BRFSS items: 1) the participant has serious difficulty walking or climbing stairs; 2) the participant has difficulty dressing or bathing; and 3) because of a physical, mental, or emotional condition, the participant has difficulty doing errands alone, such as visiting a doctor’s office or shopping. Respondents who answered yes to at least 1 item were coded as having a limitation. Respondents who answered no to all 3 items were coded as not having a limitation. This composite measure is consistent with prior studies using BRFSS disability and activity limitation items to identify functional and mobility limitations (19).

Nonmodifiable demographic covariates included age, race or ethnicity, and sex at birth. Age, a continuous variable in the dataset, was collapsed into 3 categories with 10-year age groups: 55 to 64; 65 to 74; and 75 or older. Race and ethnicity are more finely disaggregated in the Hawaiʻi BRFSS than in the national BRFSS, allowing for greater specificity of statistical findings that better represent the ethnically diverse population of Hawaiʻi. For instance, the US Census Bureau defines race as broad groups and ethnicity and as whether a person is of Hispanic origin, whereas the Hawaiʻi BRFSS recognizes multiple ethnic groups: “People who are Asian can identify as one or more ethnicities within the Asian race category; examples include Japanese, Chinese, Korean, Vietnamese, Filipino, Thai and many others” (20). Participants in this study were from the 4 largest racial and ethnic groups in Hawaiʻi: White, Native Hawaiian, Filipino, and Japanese. Respondents were included if they reported 1 of these categories as their only race or ethnicity or if they indicated it was their preferred racial or ethnic identity. Sex at birth included female and male.

Additional covariates were included as indicators of SDOH potentially associated with limitation status. These included modifiable economic covariates: education level (high school or less; some college or technical school; college or more); health care coverage defined in BRFSS as having any form of health insurance, including prepaid plans and government-sponsored coverage, including Medicare or Medicaid (yes or no); and household federal poverty level (FPL), calculated by using imputed data by the Hawaiʻi Health Data Warehouse (0% to 100%; ≥101%; unknown) (21). Also included were neighborhood walkability (yes; no; unknown); marital status (married or partnered; not married or partnered); and island of residence (Oʻahu; other island). Neighborhood walkability was derived from the BRFSS physical activity environment question assessing the built environment. Respondents were asked whether there were sidewalks, walking paths, or other places to walk near their home.

### Data analysis

Data were analyzed by using SPSS version 29.0 (IBM Corp), following CDC-recommended weighting procedures to account for the complex survey design (18,22). To produce population-representative estimates for Hawaiʻi, we applied the final sampling weight and accounted for stratification and clustering in all statistical analyses. Weighted descriptive statistics were calculated to summarize participant characteristics for the overall sample. Participants were then categorized by mobility limitation status (no limitation vs ≥1 limitation), and weighted bivariate associations between limitation status and demographic and SDOH variables were assessed by using design-adjusted χ^2^ tests that account for the complex BRFSS sampling design. To maintain compliance with Hawaiʻi Department of Health data reporting standards, variables with fewer than 50 unweighted respondents within any racial or ethnic subgroup were suppressed.

All prevalence and regression analyses accounted for the survey design. Weighted prevalence estimates and corresponding 95% CIs for mobility limitations were calculated for the total sample and each racial or ethnic group. These estimates were used to compare prevalence of mobility limitations across racial or ethnic groups and to evaluate their associations with SDOH indicators. Finally, multivariable logistic regression analyses were conducted to calculate weighted odds ratios (ORs) and 95% CIs for SDOH variables and mobility limitations. Adjusted ORs were estimated for the overall sample and separately within each racial or ethnic group. Adjusted models controlled for relevant covariates to provide more accurate estimates of the associations between mobility limitations and the independent variables. A *P* value < .05 was considered significant.

## Results

### Sample characteristics

In the weighted sample, 38.1% of respondents were aged 55 to 64, 35.9% aged 65 to 74, and 25.9% aged 75 or older (Table 1). More than half (53.7%) of respondents were female, and 62.6% were married or partnered. Regarding education, 33.5% of respondents had a high school education or less, 35.4% had some college or technical school, and 31.2% had a college or technical degree. Most respondents, 75.3%, reported household income greater than 100% of the FPL, and 97.7% had health insurance. Respondents tended to live in walkable neighborhoods (55.9%) and on the island of Oʻahu (62.4%). 

**Table 1:** Characteristics of the Total Sample in a Study of Mobility Loss, by Race and Ethnicity, Hawaiʻi Behavioral Risk Factor Surveillance System, 2019–2021^a^

Characteristic	Total sample		Raw N (weighted %), by race and ethnicity				*P* value
	Raw N	Weighted N	White	Filipino	Japanese	Native Hawaiian	
**Total sample**	10,039 (100.0)	350,922 (100.0)	5,429 (38.3)	1,112 (18.2)	2,205 (31.3)	1,293 (12.2)	NA
**Mobility limitation**							
Difficulty walking	1,755 (17.5)	60,662 (17.3)	888 (16.3)	177 (15.5)	356 (16.2)	334 (25.8)	<.001
Difficulty dressing	354 (3.5)	12,336 (3.5)	189 (3.8)	36 (3.4)	65 (2.7)	64 (4.8)	
Difficulty with errands	660 (6.6)	23,300 (6.6)	352 (6.7)	78 (6.4)	125 (5.6)	105 (9.6)	
All combined	1,980 (19.7)	68,412 (19.5)	1,006 (18.7)	218 (19.0)	391 (17.3)	503 (28.3)	
**Age range, y**							
55–64	3,393 (33.8)	133,865 (38.1)	1,692 (35.5)	484 (46.0)	631 (33.3)	586 (47.0)	<.001
65–74	4,124 (41.1)	126,094 (35.9)	2,370 (39.0)	404 (33.2)	903 (35.9)	447 (30.5)	
≥75	2,522 (25.1)	90,963 (25.9)	1,367 (25.5)	224 (20.8)	671 (30.7)	260 (22.5)	
**Sex at birth**							
Male	4,512 (46.3)	162,609 (46.3)	2,559 (50.8)	428 (38.7)	979 (45.7)	546 (45.4)	<.001
Female	5,527 (53.7)	188,313 (53.7)	2,870 (49.2)	684 (61.3)	1,226 (54.3)	747 (54.6)	
**Education**							
High school education or less	2,493 (24.8)	117,509 (33.5)	983 (26.1)	490 (52.1)	425 (24.5)	595 (52.0)	<.001
Some college/technical school	3,005 (29.9)	124,074 (35.4)	1,597 (35.8)	330 (31.3)	659 (37.6)	419 (34.4)	
College/technical degree or more	4,541 (45.2)	109,340 (31.2)	2,849 (38.1)	292 (16.7)	1,121 (37.9)	279 (13.6)	
**Health insurance**							
Yes	9,801 (97.6)	342,969 (97.7)	5,303 (97.8)	1,074 (96.9)	2,169 (98.4)	1,255 (96.9)	<.001
No	238 (2.4)	7,954 (2.3)	126 (2.2)	38 (3.1)	36 (1.6)	38 (3.1)	
**Household FPL**							
0%–100%	1,101 (11.0)	42,743 (12.2)	467 (8.3)	267 (27.3)	122 (6.1)	245 (17.2)	<.001
≥101%	7,751 (77.2)	264,102 (75.3)	4,337 (79.3)	703 (60.1)	1,799 (81.1)	912 (70.3)	
Unknown	1,187 (11.8)	44,077 (12.6)	625 (12.4)	142 (12.6)	234 (12.7)	136 (12.6)	
**Walkable neighborhood**							
Yes	5,141 (51.2)	196,050 (55.9)	2,594 (52.9)	555 (52.4)	1,342 (63.0)	650 (52.0)	<.001
No	4,135 (41.2)	125,111 (35.7)	2,492 (40.2)	396 (33.1)	715 (30.3)	532 (38.7)	
Unknown	763 (7.6)	29,761 (8.5)	343 (6.9)	161 (14.4)	148 (6.7)	111 (9.3)	
**Marital status**							
Married/partnered	5,686 (56.6)	219,504 (62.6)	3,079 (62.5)	696 (65.4)	1,242 (63.4)	669 (56.4)	<.001
Not married/ partnered	4,353 (43.4)	131,419 (37.4)	2,350 (37.5)	416 (34.6)	963 (36.6)	624 (43.6)	
**Island**							
O‘ahu	4,305 (42.9)	218,801 (62.4)	1,819 (51.4)	553 (66.7)	1,379 (75.2)	554 (57.3)	<.001
Other	5,734 (57.1)	132,121 (37.6)	3,610 (48.6)	559 (33.3)	826 (24.8)	739 (42.7)	

Abbreviations: FPL, federal poverty level; NA, not applicable.

a Percentage may not add to 100 because of rounding.

### Prevalence of mobility limitations

Prevalence of mobility limitations was highest among Native Hawaiian adults at 28.3% and lowest among Japanese adults at 17.3% (Table 2). After adjusting for sociodemographic and SDOH covariates, Filipino adults had lower odds of mobility limitations compared with White adults (AOR, 0.79; 95% CI, 0.78–0.81). Native Hawaiian adults had higher adjusted odds of mobility limitations than White adults (AOR, 1.40; 95% CI, 1.38–1.42) (Table 3).

**Table 2:** Weighted Prevalence of Respondents With at Least 1 Mobility/Functional Limitation, by Race and Ethnicity, Hawaiʻi Behavioral Risk Factor Surveillance System, 2019–2021

Characteristic	Total sample (95% CI)	White (95% CI)	Filipino (95% CI)	Japanese (95% CI)	Native Hawaiian (95% CI)	*P* value
**Total sample**	19.5 (19.4–19.6)	—^ a^	—^ a^	—^ a^	—^ a^	—^ a^
**Race or ethnicity**						
White	18.7 (18.6–18.8)	—^ a^	—^ a^	—^ a^	—^ a^	<.001
Filipino	19.0 (18.9–19.2)	—^ a^	—^ a^	—^ a^	—^ a^	
Japanese	17.3 (17.2–17.4)	—^ a^	—^ a^	—^ a^	—^ a^	
Native Hawaiian	28.3 (28.0–28.5)	—^ a^	—^ a^	—^ a^	—^ a^	
**Age range, y**						
55–64	14.9 (14.8–15.0)	15.5 (15.3–15.7)	13.1 (12.8–13.3)	8.0 (7.8–8.2)	28.9 (28.5–29.2)	<.001
65–74	18.1 (18.0–18.3)	17.4 (17.2–17.6)	19.1 (18.8–19.4)	16.5 (16.3–16.7)	24.6 (24.2–25.0)	
≥75	28.1 (27.9–28.3)	25.2 (24.9–25.4)	32.1 (31.7–32.6)	28.4 (28.1–28.7)	32.0 (31.4–32.5)	
**Sex at birth**						
Male	17.8 (17.7–17.9)	16.8 (16.7–17.0)	14.7 (14.5–15.0)	17.0 (16.8–17.2)	27.3 (26.9–27.7)	<.001
Female	21.0 (20.9–21.1)	20.7 (20.5–20.9)	21.8 (21.5–22.0)	17.6 (17.4–17.7)	29.0 (28.7–29.4)	
**Education**						
High school education or less	27.1 (27.0–27.3)	28.4 (28.2–28.7)	23.1 (22.8–23.4)	26.7 (26.4–27.0)	31.7 (31.4–32.1)	<.001
Some college/technical school	18.7 (18.5–18.8)	19.4 (19.2–19.6)	16.7 (16.4–17.0)	16.5 (16.3–16.7)	24.9 (24.5–25.3)	
College/technical degree or more	12.2 (12.1–12.3)	11.5 (11.3–11.6)	10.7 (10.4–11.1)	12.0 (11.8–12.2)	23.4 (22.8–24.0)	
**Health insurance**						
Yes	19.6 (19.5–19.7)	18.9 (18.8–19.0)	19.4 (19.2–19.6)	17.4 (17.3–17.5)	28.1 (27.8–28.3)	<.001
No	14.0 (13.6–14.5)	10.9 (10.3–11.6)	—b	—b	—b	
**Household FPL **						
0%–100%	30.6 (30.3–30.8)	43.4 (42.9–44.0)	18.5 (18.2–18.9)	26.9 (26.3–27.5)	43.1 (42.5–43.8)	<.001
≥101%	16.8 (16.7–16.9)	15.7 (15.6–15.8)	16.4 (16.2–16.6)	15.9 (15.8–16.1)	23.6 (23.3–23.9)	
Unknown	25.0 (24.8–25.2)	21.5 (21.1–21.8)	32.7 (32.1–33.3)	21.3 (20.9–21.7)	33.9 (33.2–34.6)	
**Walkable neighborhood**						
Yes	18.2 (18.1–18.3)	18.0 (18.0–18.2)	18.5 (18.3–18.8)	15.4 (15.3–15.6)	27.1 (26.7–27.4)	<.001
No	21.3 (21.2–21.5)	19.2 (19.0–19.4)	19.1 (18.8–19.4)	23.0 (22.8–23.3)	27.9 (27.5–28.3)	
Unknown	20.3 (20.0–20.7)	21.7 (21.2–22.2)	20.8 (20.3–21.3)	9.2 (8.8–9.6)	36.4 (35.6–37.3)	
**Marital status**						
Married/partnered	15.1 (15.0–15.2)	14.2 (14.0–14.3)	14.8 (14.7–15.0)	13.9 (13.7–14.0)	22.0 (21.7–22.3)	<.001
Not married/ partnered	26.9 (26.8–27.0)	26.3 (26.1–26.5)	26.9 (26.6–27.3)	23.2 (23.0–23.5)	36.4 (36.0–36.8)	
**Island**						
O‘ahu	19.8 (19.7–19.9)	19.0 (18.8–19.2)	20.0 (20.8–20.2)	17.4 (17.3–17.6)	29.5 (29.2–29.8)	<.001
Other	19.1 (18.9–19.2)	18.4 (18.3–18.6)	17.7 (16.9–17.4)	17.0 (16.7–17.2)	26.6 (26.2–27.0)	

Abbreviations: — , not applicable; FPL, federal poverty level.

a To maintain compliance with Hawaiʻi Department of Health data reporting standards, variables with fewer than 50 unweighted respondents within any racial or ethnic subgroup were suppressed.

b Estimates were suppressed because the unweighted cell size was fewer than 50 respondents.

**Table 3:** Adjusted Odds Ratios for at Least 1 Mobility or Functional Limitation in the Total Sample and by Race and Ethnicity, Hawaiʻi Behavioral Risk Factor Surveillance System, 2019–2021

Characteristic	Total sample		White		Filipino		Japanese		Native Hawaiian	
	AOR^a^ (95% CI)	*P* value	AOR^a^ (95% CI)	*P* value	AOR^a^ (95% CI)	*P* value	AOR^a^ (95% CI)	*P* value	AOR^a^ (95% CI)	*P* value
**Race/ethnicity**										
White	1 [Reference]									
Filipino	0.79 (0.78–0.81)	<.001	—		—		—		—	
Japanese	0.86 (0.85–0.88)	<.001	—		—		—		—	
Native Hawaiian	1.40 (1.38–1.42)	<.001	—		—		—		—	
**Age range, y**										
55–64	1 [Reference]		1 [Reference]		1 [Reference]		1 [Reference]		1 [Reference]	
65–74	1.32 (1.30–1.33)	<.001	1.21 (1.18–1.23)	<.001	1.39 (1.36–1.44)	<.001	2.37 (2.31–2.43)	<.001	0.82 (0.80–0.85)	<.001
≥75	2.01 (1.99–2.04)	<.001	1.74 (1.70–1.78)	<.001	2.43 (2.36–2.50)	<.001	4.05 (3.94–4.16)	<.001	1.11 (1.07–1.15)	<.001
**Sex at birth**										
Male	1 [Reference]		1 [Reference]		1 [Reference]		1 [Reference]		1 [Reference]	
Female	1.06 (1.05–1.07)	<.001	1.12 (1.10–1.14)	<.001	1.40 (1.37–1.44)	<.001	0.85 (0.84–0.87)	<.001	1.01 (0.98–1.03)	.71
**Education**										
High school education or less	1 [Reference]		1 [Reference]		1 [Reference]		1 [Reference]		1 [Reference]	
Some college/technical school	0.69 (0.68–0.70)	<.001	0.65 (0.64–0.67)	<.001	0.73 (0.71–0.75)	<.001	0.60 (0.59–0.62)	<.001	0.82 (0.80–0.85)	<.001
College/technical degree or more	0.46 (0.46–0.47)	<.001	0.39 (0.38–0.40)	<.001	0.46 (0.44–0.48)	<.001	0.48 (0.47–0.50)	<.001	0.81 (0.78–0.84)	<.001
**Health insurance**										
Yes	1.79 (1.72–1.86)	<.001	2.45 (2.28–2.63)	<.001	2.70 (2.44–2.98)	<.001	1.59 (1.45–1.73)	<.001	1.10 (1.03–1.18)	.008
No	1 [Reference]		1 [Reference]		1 [Reference]		1 [Reference]		1 [Reference]	
**Household federal poverty level **										
0%–100%	1 [Reference]		1 [Reference]		1 [Reference]		1 [Reference]		1 [Reference]	
≥101%	0.59 (0.58–0.60)	<.001	0.33 (0.32–0.34)	<.001	1.03 (1.00–1.06)	.03	0.87 (0.84–0.90)	<.001	0.49 (0.47–0.51)	<.001
Unknown	0.81 (0.79–0.82)	<.001	0.40 (0.39–0.42)	<.001	1.94 (1.87–2.01)	<.001	1.13 (1.08–1.18)	<.001	0.62 (0.60–0.65)	<.001
**Walkable neighborhood**										
Yes	1 [Reference]		1 [Reference]		1 [Reference]		1 [Reference]		1 [Reference]	
No	1.16 (1.15–1.18)	<.001	1.07 (1.05–1.09)	<.001	1.01 (0.98–1.04)	.54	1.42 (1.39–1.45)	<.001	1.10 (1.07–1.13)	<.001
Unknown	1.04 (1.02–1.06)	<.001	1.08 (1.05–1.12)	<.001	1.08 (1.04–1.12)	<.001	0.47 (0.45–0.50)	<.001	1.62 (1.55–1.69)	<.001
**Marital status**										
Married/partnered	0.58 (0.58–0.59)	<.001	0.61 (0.60–0.62)	<.001	0.61 (0.60–0.63)	<.001	0.62 (0.61–0.63)	<.001	0.57 (0.55–0.58)	<.001
Not married/ partnered	1 [Reference]		1 [Reference]		1 [Reference]		1 [Reference]		1 [Reference]	
**Island**										
O‘ahu	1 [Reference]		1 [Reference]		1 [Reference]		1 [Reference]		1 [Reference]	
Other	0.86 (0.85–0.87)	<.001	0.88 (0.86–0.89)	<.001	0.83 (0.81–0.86)	<.001	0.90 (0.88–0.92)	<.001	0.82 (0.80–0.85)	<.001

Abbreviations: — , not applicable; AOR, adjusted odds ratio.

a The overall model was adjusted for race or ethnicity, age, sex at birth, education, health insurance, household income, neighborhood walkability, marital status, and island of residence. Stratified models were adjusted for age, sex at birth, education, health insurance, household income, neighborhood walkability, marital status, and island of residence.

For all racial and ethnic groups, prevalence of mobility limitations was highest among adults aged 75 or older compared with younger adults, and higher for women than men, with the exception of Japanese women. Those who were aged 75 or older had the greatest odds of mobility limitations across all racial and ethnic groups, as well. Odds ratios were highest among Japanese adults who were aged 75 years or older, who were 4 times more likely (AOR, 4.05; 95% CI, 3.94–4.16) to report mobility limitations than Japanese adults aged 55 to 64 years. In stratified adjusted models, the association between age and mobility limitation differed for Native Hawaiian adults from the pattern observed in the other groups; compared with Native Hawaiian adults aged 55 to 64 years, those aged 65 to 74 years had lower adjusted odds of limitation (AOR, 0.82; 95% CI, 0.80–0.85), whereas those aged 75 years or older had slightly higher odds (AOR, 1.11; 95% CI, 1.07–1.15). Women had greater odds of mobility limitations compared with men among White and Filipino adults. However, Japanese women had lower adjusted odds of mobility limitations than Japanese men (AOR, 0.85; 95% CI, 0.84–0.87). The association between sexes and mobility limitation among Native Hawaiian adults was not significant.

Education and income were associated with mobility limitations across racial and ethnic groups. As education increased, the odds for mobility limitations decreased across all racial and ethnic groups. However, the reduction in odds was much less pronounced among Native Hawaiian adults, with ORs of 0.82 for those who completed some college or technical school and 0.81 for those with college or technical school completion, compared with those with a high school education or less. In comparison, White adults with some college or technical school had 65% lower adjusted odds (AOR, 0.65; 95% CI, 0.64–0.67), and respondents who completed college or technical school or more had significantly lower odds of mobility limitation compared with those with a high school education or less (AOR, 0.39; 95% CI, 0.38–0.40) (Table 3).

Mobility limitation prevalence was higher among respondents living in households with income at or below 100% of the FPL than among those with income at or above 101% of the FPL. For example, prevalence was 43.4% among White respondents and 43.1% among Native Hawaiian respondents in households at or below 100% of the FPL, compared with 15.7% and 23.6%, respectively, among those in households at or above 101% of the FPL (Table 2). In adjusted models, income at or above 101% of the FPL was associated with lower odds of mobility limitations among White, Japanese, and Native Hawaiian respondents.

Those who were married or partnered had lower odds of mobility limitations than those who were not married or partnered across all racial and ethnic groups. Married or partnered Native Hawaiian respondents had lower adjusted odds of mobility limitations than respondents who were not married or partnered (AOR, 0.57; 95% CI, 0.55–0.58). In addition, a notable geographic pattern emerged: residing on an island other than Oʻahu was associated with lower adjusted odds of mobility limitation across the 4 racial and ethnic groups.

## Discussion

We found that disparities in mobility/functional limitations — defined by difficulty walking, climbing stairs, dressing, bathing, or running errands independently — exist in community-dwelling older adults (aged 55 years or older) by racial and ethnic group in Hawaiʻi. Native Hawaiian adults had the highest prevalence of mobility limitations, and disparities were already pronounced among adults aged 55 to 64 years. These findings support those of Pobutsky et al’s 2003 analysis of Hawaiʻi BRFSS data that found that Native Hawaiians aged 65 years or older were more likely than White and Japanese adults to have activity limitations. Together, these findings suggest that disparities may emerge before age 65 years among Native Hawaiian adults in Hawaiʻi (8).

After adjusting for SDOH covariates, Native Hawaiians had the highest odds of experiencing mobility limitations when compared with the other racial or ethnic groups in the study. Associations between selected SDOH indicators and mobility limitations differed across racial and ethnic groups. For example, education acted as a protective factor in the sample, supporting findings from other research that has explored the association between education and mobility (23,24). The protective association of education appeared weaker among Native Hawaiian respondents than among White, Filipino, and Japanese respondents. Having a household income over 100% of the FPL also showed a reduction in risk for mobility limitations. Respondents with health insurance had higher odds of reporting mobility limitations across groups. The most plausible explanation is reverse causality: adults experiencing functional or mobility limitations may be more likely to obtain or maintain health insurance coverage because of increased health care needs. 

The association between mobility limitations and social variables followed patterns seen in other SDOH health research (25), with respondents who did not live in walkable neighborhoods and respondents who were not married or partnered having higher odds of mobility limitations. Although household income and neighborhood walkability were significantly associated with mobility limitations, the relatively large “unknown” categories for income and neighborhood walkability limit interpretation of those associations.

Respondents living on islands other than Oʻahu had lower adjusted odds of mobility limitations than those living on Oʻahu. This may be because of the higher activity levels in nonurban spaces that act as a protective factor against developing mobility limitations. It could also be that residents with mobility limitations relocate to urban Honolulu because of the limited social services on the neighbor islands. These include transportation, in-home chores, and food delivery services that can help older adults with mobility limitations to age in their homes (26).

The assessment of mobility limitations in clinical settings is routine for geriatric patients. However, with Native Hawaiians experiencing mobility limitations at higher rates and at younger ages, waiting until age 65 years to assess mobility limitations may miss an opportunity for earlier identification and intervention. Mobility assessments are effective at identifying mobility deficits when they are already present; however, identifying emerging mobility limitations from cardiac, respiratory, or vascular issues requires an assessment of movement over a longer distance. A study with older adults aged 70 to 79 years found that endurance walk tests were helpful in early detection of mobility deficits (27). Educating older adults and their families on signs of mobility limitations, such as decreasing ability to walk a quarter mile, may help in the identification of mobility problems outside of the clinical setting that then can be reported to the care team during routine visits.

### Limitations

This study has several limitations. Results and interpretations made from the dataset may be inaccurate without the context of the lived experience of older adults who completed the survey. The BRFSS is a cross-sectional survey, which means that longitudinal analyses could not be conducted, and conclusions on causation could not be made. Findings are limited to the racial and ethnic groups included in the analysis and should not be generalized to all adults in Hawaiʻi.

The study was limited to the mobility questions posed by the BRFSS questionnaire, which includes nationally standardized sections and items. Although it is possible for researchers to add questions to state-specific questionnaires, more refined mobility questions were not included in this survey in Hawaiʻi from 2019 through 2021. In addition, the item on difficulty doing errands alone because of a physical, mental, or emotional condition extends beyond physical mobility alone. As potential confounding factors related to mental health were not fully accounted for in the analysis, this composite outcome should therefore be interpreted cautiously.

The data were collected during 2019 through 2021, which spanned the peak of the COVID-19 pandemic. The COVID-19 pandemic came with strict quarantine policies, particularly in Hawaiʻi, that reduced people’s ability to exercise and socialize. This may have reduced opportunities for physical activity and community participation among older adults or exacerbated pre-existing mobility disparities. However, the BRFSS dataset does not allow direct assessment of pandemic-related behavioral changes; this should therefore be considered contextual background rather than a study finding.

### Conclusion

This study identified significant racial and ethnic disparities in mobility limitations among community-living adults aged 55 years or older in Hawaiʻi. Native Hawaiian adults had the highest prevalence and the higher adjusted odds of mobility limitations, with disparities already evident among adults aged 55 to 64 years. SDOH, particularly income, education, and marital status, were strongly associated with mobility status across groups, although the protective effects of these factors were less pronounced among Native Hawaiian adults.

These findings have clinical and public health implications. Earlier onset of mobility limitations suggests that waiting until the traditional Medicare eligibility age of 65 to screen for mobility impairment may miss an important window of prevention. Clinical practice and community health programs in Hawaiʻi may benefit from considering mobility screening and prevention strategies earlier than age 65 years, particularly for populations at elevated risk.

Tailored interventions are also needed to address inequities in functional aging. Culturally grounded and community-based mobility programs, improved access to supportive services, and policies addressing SDOH may help reduce disability risk and support aging in place. Prioritizing early, culturally tailored mobility screening and support for Native Hawaiian adults well before age 65 may mitigate some of the disparities observed in this study.
